# Odontological Society of Pennsylvania

**Published:** 1890-02

**Authors:** W. G. A. Bonwill

**Affiliations:** Philadelphia


### Reports of Society Meetings.

### ODONTOLOGICAL SOCIETY OF PENNSYLVANIA.

### RECOLLECTIONS AND IMPRESSIONS OF MY VISIT TO THE AMERICAN DENTAL SOCIETY OF EUROPE, AT PARIS; BRITISH DENTAL ASSOCIATION, AT BRIGHTON; AND THE FIRST INTERNATIONAL DENTAL CONGRESS, AT PARIS.[1]

BY W. G. A. BONWILL, D.D.S., PHILADELPHIA.

As your minister accredited to these foreign courts, it is expected that I shall relate whatever of interest came under my observation.

It is very proper that delegations should be sent on all state occasions, in order that an estimate may be drawn of just how nearly related we are to those who are so infrequently in communion with us. Nothing else has promoted the well-being of professions, trades, and societies so much as the close relations of intimacy gained only by personal contact with these great bodies.

That we, especially, should know our brothers across the water more intimately, as a body, is evident, for it would promote a better feeling than now exists towards "American Dentistry," and materially enhance and elevate the broad standard of dentistry which is so needed to give all a higher social status among ourselves and among the classes for whom we operate. The dental journals have carried every phase of work and thought from all parts to every hamlet of the civilized globe, yet I know, from this summer's experience, that we are far from that goal which every true man could desire,—the status vouchsafed to all the other learned professions,—and which we cannot hope to reach until we can learn to treat each other individually and collectively as is becoming gentlemen; until we can learn to estimate a man by what he has done, and extend to him the honor due him; until we can forget that science knows no nationality; then dentistry will succumb to the inevitable, and divest herself of her swaddling-clothes as a trade. I did not know when I left here the animus against the "American dentist;" but it did not take me long to sniff it when I reached England.

Personally, I was treated with the greatest consideration.

Everything was done to make my visit honorable and pleasant. At my clinics, at Brighton, the crowd around me was dense, and I was told—in fact, I could see and feel the spirit animating nearly every one—that no closer attention to my speeches nor demonstrations could have been given. To see such an anomaly as packing—welding—Abby's soft foil with smooth oval points was beyond belief, and they were as incredulous as my own countrymen after twenty years' teaching.

When I attempted to demonstrate the extraction of a tooth by rapid breathing the crowd was so great as to overpower my patient with fright, and he could not rapidly breathe. But I demonstrated it upon myself, to their satisfaction, by their pulling my beard and hair, pinching, and placing finger on the conjunctiva of my eye.

But, aside from all this personal respect and attention given me, I saw clearly that when the reports were made from the dailies, no mention was made of my operations, nor of me further than that "Dr. Bonwill, a leading American dentist, was present at the meetings." Another thing: before I went to Brighton, I found that my clinics were not down on the manuscript programme, although I had stated in advance that I would be present, and would assist in every way I could to make the meeting profitable. It was finally added.

What first led me to suppose that there was an undercurrent of feeling against the "American dentist" was, when I proposed to go into the American Dental Institute, a branch of which was at Brighton, in order that I could see what American dentistry was doing to honor its country, I was begged not to do so, as the next day there would appear in the circular, published regularly by the Institute, "That the celebrated Dr. ——, an American dentist, had honored it with a call;" and it thereby would give status to its nefarious business and work to the detriment of the English dentist.

This American Institute, I learned, had several branches, and was run by a Jew who was not a dentist, but employed many young American dentists to give the most approved style of operations in every department; and they resorted to extensive advertising and special circulars as operations done only by the "advanced American dentist." Not wishing to give offence, I did not enter their portals. Notwithstanding the novelty of all I had demonstrated, this treatment could but confirm me now in my conjecture that I must suffer silent praise on account of the outrageous conduct of many men who were posing as American dentists.

To make it still more noticeable, I found that I was not down on the banquet programme for a speech in reply to the toast of "Visitors." Now after such personal attention by the best men in London, and at a banquet given by the dentists alone of Brighton, where I had already received the most honored seat, I was somewhat doubtful about accepting the invitation to the grand banquet. It was so very noticeable, I could but feel that there was some mistake, and that it would be corrected at the last moment. This was not done. At the banquet they gave me, next the officers, the most honored seat. All went off well until the visitors were called upon to reply to the toast. The French delegates were, with another visitor, called upon,—none of whose names were down for a speech on the programme; but my name was not called by the toastmaster. At this juncture I was boisterously called for by a large number. It was an embarrassing moment to me. I could feel that the affair was intended to be not an insult to me, but a silent rebuke, at my expense, to the "American dentist."

I had already made a few remarks, at the opening of the meeting, extremely complimentary to the English dentists: That I was pleased to meet them; and, while I came to drink from their fountain of experience and knowledge, I should feel that my visit had been in vain if I could not give some recompense in what I would demonstrate of the class of operations I was personally doing in my office. This was very palatable to them, and yet it was not noticed publicly, except as a "characteristic American speech." At the banquet, to be sure that it was not aimed at me, I, while again using quite the same language and sentiments as previously made, spoke of what I had seen already in England, Germany, and France, as to the materials used, finding that plastics were the rule and gold the exception; and added, that *we* in America had concluded that there was nothing so well calculated to give to every operator such dexterity in manipulating plastics as excelling in the use of gold in contour work under both hand-pressure and the mallet. By the use of "we" I thought I would be sure to take the burthen off my own shoulders and perhaps please them by the greater authority than myself, my honored countrymen. This "we," however, brought things to a crisis, as I learned before I retired that night. One of England's honored Sirs told me that had I spoken for myself alone all would have been well, as they well understood what my character and standing was in their country.

This feeling was not universal, for many men came to me after my remarks and thanked me for my gentle touch. While very unpleasant for me at the moment, I did not regret that the blunder was made, for it brought to the surface what was already at fever heat; but the English dentist was too proud to speak of it, unless some good opportunity should offer. I am glad to have been the means of their laying before me, in all its blackness, the doing of the "American dentist" abroad. I shall expose these to you to-night in order that something may be done to make a radical change, so as to blot from the European escutcheon the ignoble title of "American dentist."

To make my position impregnable as a bearer of despatches to you from the dentists of Europe, I wrote to many of the leading practitioners in England, France, and Germany, and they were unanimous in denouncing the average American dentist as found among them. I need quote but from one of the best men in London. He said, "Then, there is another thing. We are accustomed to have everything possible made use of and perverted for their own purposes, by advertising-folks, like the American Dental Institute; and it was thought—I do not know as I said it before of my own knowledge—that your speech contained some plums for them—this Institute—to pick out and use."

This tells in unmistakable language what I had to bear for my countrymen, because of the dishonorable and disreputable men from America. There are others, too, who come from England, as well as from all the European countries, for the sole purpose of returning and placing on their signs the reputable and honored name, "American dentist," many of whom cannot speak one word of English.

The blunder of simply saying "we," while making me the target, also gave me the high privilege of being the exponent of the aggrieved, and I shall feel amply rewarded for the unpleasant position in which it placed me, if the schools of this country grow more careful in the class of students and the restrictions they place upon those intending to practise abroad. I was asked to lay this matter before my society, and have it agitated in order that this condition of affairs may soon terminate. To go on would place a veto on all international congresses, whether associated with the medical or as dental only. Were another congress to be held in London or England, Americans would not have a right to expect the courtesy which was so extensively lavished upon them in 1881.

The noble movement of the dentists of England then, in gaining for us a recognition in the "International Medical Congress" for the first time, entitles them to the highest consideration and thanks. Everything is being done in their schools to give that social status to the dentist which is enjoyed by even the average medical man. They are more careful than we, by far, about the higher general education and men who have already better social standing. But they feel that their work is largely in vain when we are turning out such vast numbers to flood the market and sail under the banner of "American dentist."

While the average Englishman is a free-trader, yet, when it comes to the professions, he at once sees that it is unfair and unprofitable. Free thought they advocate, but not the freedom to force ignorance and ordinary ability upon them under the name of "American dentist." That they honor individual Americans for much of the advancement and progress of the last quarter of a century, and adopt the methods and practice of any of us who have proven it to be best, I have the honor to know, and the name of such is never withheld.

Until this assumption of one country over another as to being first can be settled to the honor of all, we need not expect that such parading will add to the character of dentists before the world. While America has justly the right to the title and honor of leading in the race of practical dentistry, we need not feel that it is because we have any better material to make dentists of. I found as bright men wherever I went as we have at home. But, gentlemen, circumstances very much alter the results. We have had the widest field for development in the immense destruction of teeth from our habits, with more general distribution of wealth to pay for operations, while in Europe it is the reverse. How few dentists are to be found in Germany, France, or England compared with the number in America! Besides, as dentists become better educated and are brought from the ranks of such as can be made gentlemen, and who value the tone and air that hang around the true professional man, we shall find fewer turned out than now.

At present there are more dentists than have a right to legitimate business, unless they will turn their attention to the poorer classes who are in such need of the competent operator. I can see that competition must come to the rescue or the poor will go without their teeth being preserved. Artificial teeth will be the universal degradation of the poor, and no effort will be made to rescue the living organs. I had many examples of high-toned American dentists pointed out to me who had given no cause for offence, and who had at the same time made large practices without having advertised or spoken against the native talent.

I do not wonder that the American dentist should have such bad repute, and the feeling against the American physician is just as high. Germany and Switzerland particularly are growing more intolerant every year. When you consider how impoverished the average medical man or dentist is in any of the European countries, you can well understand that self-preservation must step to the front. If we could make the operations cheaper and better, and could reach thereby the poorest, who need our services as well, we might have the American push on.

One strong point I must make against my countryman is that, when he goes abroad as an American dentist, he does not hold up the standard of dentistry as taught here, but at once falls into the same rut as the native dentist. Many of those I met abroad confessed to me that they would starve if they did not give up and cater to the desires of those who had been educated to a certain standard! Few plates of partial sets are made without clasps, and the old gold spiral springs, which are seldom used in this country, are still in universal favor wherever I visited. To attempt to make them without would be suicidal to even the American. Instead of extracting every root, as we do here, all are retained and the plate placed in over them.

I did find one exception, in my student at Leipsic,—a graduate of the University of Pennsylvania, whom I taught my plan of articulation. He has braved it because he has a remedy by which he can do without the spring. When this is generally understood and practised the rule will change. If our men would follow the line of practice taught them, they could more honorably place on their cards, not "American dentist," but "Graduate of an American school."

Again, they take advantage of their own inability to perform good and lasting fillings, and resort to crowning in every instance, where we know that amalgam, and even gold, and for less money, could have been used. The reckless destruction of splendid teeth by crown- and bridge-work is something too awful to contemplate. I have seen, not only in Europe but at home, teeth crowned that could have been preserved for ten or fifteen years with amalgam, and often with gold. I heard one of our American boys, on the other side, boasting of the character of his practice, which had almost been revolutionized since crowning came into vogue, and he was doubling his income.

Now, while the average American has acted so unprofessionally abroad, I can see that his mission has aroused the native dentist to attempt more than he ever did before. His energy has been doubled, and it has urged him on to study and to fly for graduation to some school at home or abroad; and thus the American has been a blessing in disguise.

On the whole we cannot blame the American so much, for he has only gone, just as the Englishman, where the English language is spoken. This thing will correct itself in time. The schools in England, France, and Germany are doing a good work, and are holding on to many students who once would have sought our shores. In the University at Berlin I found, under Professor Miller, two hundred students. When I gave a clinic with gold and smooth points in my mechanical mallet, they piled up around me as if they were greedy to see and profit thereby. They were full of enthusiasm. The professor of mechanical dentistry gave me especial attention when I demonstrated my articulator and its laws. He sat up all night to arrange a set such as I had exhibited, so determined was he to succeed before I left. This school will be a great thing for the German student, as soon as Professor Miller can have more help from demonstrators. The burden on him now is rather too much. The demands in that country cannot be great, and consequently the number of students must be for a long time rather limited as long as there are so many poor who cannot pay high prices.

In Paris there are two flourishing schools, in which there is rather too much rivalry, but no more than to be found here. The French are ready and willing to add their ability and earnestness to elevate their profession. Their enthusiasm is unbounded in whatever they undertake, and I see no reason why there should not be a central school, where, from their ingenuity and precision in all lines of work, they should make splendid dentists, who will rival any of us.

It would be impossible to give you any idea of the character of the operations done in England and the continent, as their offices are sealed books. I could judge of them only by the description from each individual. The clinics given at the Brighton meeting I could not see on account of being engaged on my own work, but they were not very extensive. The papers read on anæsthetics were fine and worthy the highest commendation, showing thorough investigation and practical experience. The mixing of oxygen and nitrous oxide was certainly of value, and should be always used, as it no doubt lessens any tendency to injury and obviates that purple hue so frightful to look upon when the gas alone is given.

These papers alone gave character to the whole proceedings and had an elevating influence. The practical part of dentistry did not have much show in extensive papers. Prosthetic dentistry was scarcely touched, and no discussion on the one paper that was read. The microscopical section was very fine, and showed great care and research.

The many abnormal and anomalous cases were not only interesting but instructive. Many plans of pivoting were shown as models, and bridge-work came in for its full share, even in England. There was one method of removable bridge which was well worthy of special mention. It was a bar anchored permanently between two teeth by either caps or amalgam in cavities of decay. This bar was narrow and allowed to be as wide or deep as possible, reaching from the gum to grinding surface, that it should prevent the teeth, which straddled the bar, from moving laterally, but allow of sufficient frictional bearing to keep the block of teeth firmly thereon. Where there was plenty of room, or rather depth or length to the crown, it was very practicable. It reminded me of Dr. Wm. C. Eastlack's plan of doing black work on metal plates.

The exhibits of manufacturers of teeth and instruments and chairs were fully equal to anything I have seen at our American Dental Association meetings. The English tooth is certainly beautiful and well made, and has all the advantages of ours, except, perhaps, it will not stand the test in soldering on account of the greater density. But for vulcanite and crown—all porcelain—we need nothing stronger or more beautiful and natural in the mouth.

The burrs given me by Ash & Sons are all cut by young women, and compare well with anything we have on this side of the water made by men. The "Dental Manufacturing Company" is not behind in anything, and proves that dentists can manage such an establishment both with profit and success, as well as manage a dental journal in the interest of dentists.

The colleges I had no chance to visit, as they were practically closed in London. While so much has been done to raise the standard, the end in view—to give to dentistry the same status as the physician—is not an accomplished fact.

I sat beside an M.D. who, with many other physicians, was a guest at the banquet, and, in conversation with him, he frankly stated that dentists were not considered on a par with physicians, although associated in the section of an International Medical

Congress. They feel towards the bulk of them their superiority, and only a favored few have the coveted social standing. I know one who was highly respected by the English dentists, yet he told me that the social status of dentistry, whether American or native, is far from gratifying.

We need not soon expect that same social position and recognition held by medical men, who have labored for years to reach it. Then, a higher educational step, such as the colleges in this country have just taken in the three years' course, will, if they have an honest preliminary examination, rapidly elevate the dentist. In England they are surely working to this end. I can see the barometric marks of elevation since I commenced in 1854. While you may turn out of your schools all that may come, instruct them, should they go abroad, not to assume the name of "American dentist," thereby implying a superiority to all other nationalities.

As I said, science is broader than nationality, and we should be a universal brotherhood, that, by our conduct to each other, we can let the world see that we are above the competition of the tradesman. This is all they ask of us abroad. So high has the feeling run against us, that the American Dental Society of Europe is frowned upon by the natives all over the continent, and it has but few to attend its gatherings.

The patent craze, in England, is not without defenders, especially when it comes to certain articles that require time, capital, and labor to perfect and put on the market, and where they are adapted to other uses than dental. Notwithstanding the number of inventions I have patented, I was not cut at, as at home, at any place or by any one. One prominent dentist of Paris, who had always opposed me, when in America, on patents, after he had read my argument in the INTERNATIONAL DENTAL JOURNAL for July, 1889, and after a talk with him and some invited scientific gentlemen on the laws of articulation and its application against evolution, asked me, "Bonwill, did you patent the articulator?" For several days I gave him no reply. He said, "If you did not, you should have done so, for it well merits a patent."

Then I feel satisfied that, if we shall have more consideration for the dental profession on the other side, and shall teach students not to use "American dentist" on their sign or specially refer to it, but simply to state the college or colleges where they have graduated, we shall bring about a change in the condition of affairs.

I would, in the mean time, advise my English friends to dash ahead and show that they can be equal to any emergency. Let them rise above such surroundings, and take the advice which I so generously gave them at the banquet, and which I gave where-ever I went on the continent, to cultivate the art of filling with gold, as I showed them at my clinics. This would not only enable them to place in more gold fillings, but, from the less time consumed by these methods, they could make equally as much money as when plastics are used. Besides, patients would bear the operation with pleasure, since gold certainly looks superior to amalgam in many places; and, as to pain, it could not be an objection, and the time consumed would not be much greater than that for some amalgam fillings or the placing on of a crown.

If they could universally change their system of fees, I think, they would command more profit and be to the advantage, in every way, of operator and patient. But whatever changes they may make, there cannot be so many teeth preserved satisfactorily unless more time is taken to wedge them apart and contour every approximal filling. If they only use amalgams and other plastics, it must be done only by getting greater space between the teeth to prevent future decay, and make it tolerable in mastication. Contouring in England and on the continent is done but seldom; nor can it be done to be of value unless, as I have said, time is taken to wedge teeth apart to prevent future caries.

Then what I said at the banquet, at Brighton, I do not regret, for it brought to the surface the drift, and I have no doubt of the result of the airing. When we look at home, we find the same spirit existing among our own countrymen. Jealousy, envy, and competition, from members alone, is rampant, and so it may ever be. We have, perhaps, as great a number of poor dentists, in proportion, as elsewhere. Higher education and better material will weed out the weaker, and by the strenuous execution of the laws we can expect that dentistry will rise or go down, as, by the laws of political economy, they must.

We should not lose sight of the ever-present fact that the poor are not to be forgotten. We cannot all be high chargers. We must let every man set a valuation on his own operations, and he who does the best need not fear. The increasing numbers will or should make it better for the world at large.

The American Dental Society of Europe I attended in Paris. They had a two days' session only. But few were present, and they mostly Americans. As I before intimated, it is not recognized by the natives of England or on the continent. It is evidently kept up as a social affair. I do not think the natives should feel too strongly towards them, for it is not done to keep out any one, no matter what his nationality. It is only a few of the natives who can travel the long distances and go to the expense, unless there could be a universal language. The many literary journals now sent to every hamlet fill largely the place of the dental society in countries where men cannot afford to travel. The illustrations and full proceedings of the principal societies in the large cities are so complete that men need not often leave their offices to know what is being done every month. Now and then clinics can be held that reinforce the mere description of difficult operations. To me it was very gratifying to meet dentists whom I had known through their writings and labors. Go where we will, each nation has its colony; socially, nothing is thought of it; professionally, they should be tolerated, if no insult is intended to the nation on whose ground they meet. It might be less objectionable to call themselves the Americo-European Dental Society. I saw as good operations in gold, amalgam, tin, tin and gold, oxyphosphate, and gutta-percha, in one mouth, in contour as well as flat surfaces, done by a German dentist as well as I could ask at the hands of any American dentist.

They did not do justice to the meeting, for there was such a rush to "do" the exposition and hurry back to their offices. The principal feature was the lantern exhibition of Professor Miller of his special work in bacteriology, which was very fine; but this can be really better studied when transferred to a journal and experiments are made in one's laboratories. The subject of gold and tin for filling was fully discussed, as it has been before, without new light from those who advocate it.

I was asked for my views as to the conservatism of filling-materials, and I had occasion for the first time to combat the views of Drs. Miller and Jenkins, who are the bulwarks of the gold and tin combination. This elicited from them the true reason why they use it so largely. 1st. Gold alone, in caries of any extent, is not only too expensive for the bulk of their patients, but they cannot afford to take the time necessary to pack in so much gold with the old methods and appliances they still use, and the patients will not put up with lengthy operations. Tin alone, while it will save, they admit, as well as the combination of gold and tin, yet, it will not stand mastication so well as the latter. 2d. In teaching at the Berlin University, the combination is almost exclusively taught over gold, for the reason that it can be more easily grasped by the average student than learning to manipulate gold by the old plan that has proven a failure.

Professor Miller and Dr. Jenkins were fair enough to say that they did not doubt my ability, with the instruments I used in clinic, especially the mechanical mallet, to do all I claimed in the use of gold; that it would perfectly preserve tooth-structure in just such cases as I advocated, as well as the combination, and more,—that perfect contour could be restored, and would resist mastication, which would be impossible with the combination. But, inasmuch as most of their operations are made flat faces with permanent separations and allowed to approach each other at the cervix, when caries does occur again it can more easily be repaired or renewed than if gold is used.

They further admitted that, with such an instrument as the mechanical mallet, with the points I am now using, it is possible to put in a large contour gold filling as quickly as when done with gold and tin. So that there is really nothing in the combination that cannot be done with gold alone, provided the operator has the ability to manipulate gold as easily and has our advantages of appliances. Then I could well understand that it was justifiable to use the combination if the patient had not the money, nor the time, nor the pride to submit, when there was a probability that gold would not last. When I placed in a large compound filling of gold at the University of Berlin, Professor Miller admitted it would take him as long to have done it with the gold and tin.

There was but one clinic given besides the one I gave in amalgam; because of an injury to my right index finger I could not make a gold filling. I found that my method of impacting amalgam with bibulous paper under heavy pressure was quite universally practised; but, strange to say, the true spirit of the procedure was not grasped. They mixed their amalgam too thin, and did not use as much pressure as would perfectly drive out the mercury. I found the same trouble in England, which arose from a misconstruction of the description given by Mr. Charles Tomes. My clinic soon convinced them that the amalgam, while it should not be squeezed dry, should be as stiff as could possibly be worked in the palm of the hand with the fingers.

To be satisfactory the meetings of the American Dental Society of Europe should never be less than three days. The social side was fully met by a splendid private entertainment given by Dr. Crane, and the banquet, which was on the Eiffel Tower, was unique and in every way a success.

I regretted to see so few of the French dentists and of the American dentists of Paris in the meetings. I think, if they would use their efforts to form local societies throughout Europe, it would have a happier and more useful effect in drawing together men who would be glad, no doubt, to gain knowledge from any source. There is not enough new material every year that is worthy of exposure, and they should have a triennial meeting when the International Medical Congress meets in Europe. This would be quite often enough, unless it be for social and mutual admiration.

I was invited to clinic and lecture in Leipsic, Germany, but my stay was too limited. This school is very well fitted up, and commands quite a class, which is largely German. The students are not taught the use of gold for filling as we understand it, for they have not the appliances. They were very desirous of seeing me operate in gold and amalgam. In fact, wherever I went they were anxious to see how we do things in this country, and they had some curiosity to see how fast I could operate, and the reason why it could be done so much quicker than by their processes. I said they are not taught at the university any but the slower methods. While this is a fact, yet it need not be so, for my student, Dr. Paul Schwartz, of Leipsic, a graduate of the University of Pennsylvania, who is demonstrator in their Mechanical Department, is well capable of teaching them every minutia as he was taught here. He has necessarily met with opposition at his own home, as our system is so radically different; yet he persists in carrying out to the letter what he knows from the chances he had in witnessing so many of my private operations. In a short time, notwithstanding the chances are largely against our methods, with such men in their midst, great changes may be expected.

It is not that the European dentists do not wish to learn and adopt the very best system; but with them reform must come slowly, since they have not the means at their command in the shape of wealthy patients. Since it is an accepted fact that gold can be packed so quickly as to overcome the serious objection to its use of consuming much time without adequate pay, and that crown- and bridge-work can be made so profitable, as perfected in this country, they must adopt it very soon or fall far to the rear.

It remains for me now to speak of the First International Dental Congress, held in Paris, September 1, 1889. As the first experiment of an International Dental Congress it was a *grand success;* and considering the opposition given it both at home and in America, and England particularly, it was marvellous that it should have had so many delegates and attendants. It was not surprising that it should have met such stanch opposition. It was a serious question whether such a step would not be sufficient ground to make the International Medical Congress, as well as the American Medical Association, refuse further to recognize us by a section in their meetings.

I am proud to have been one of its members, assembled to test whether we had the ability to stand alone. Its representation was equal to that of any congress held in Paris, and by men from every country in Europe; and even from America, where so much was done to make it a failure. As to enthusiasm, we had it from the moment the congress opened. The meetings were replete with interest and the rooms always full, notwithstanding the attractions of the Exposition and of the gayest capital of Europe.

The clinics were crowded to suffocation. Once I had to give up; the numbers were so great I was almost suffocated, so anxious were they to see everything done which was calculated to demonstrate the greatest advances of our art. Never before, on any occasion, did I see so many wild with delight at the sight of so much to carry to their homes. While the element was largely French, there was not such complete organization as could have been wished; yet, like everything done in Paris, on this their greatest *fête*, it was worthy of having been born on French soil.

It was just there where the congress should have had its birth. There, in conjunction with so much of the Old World, which needs to have had this at its own doors, we, who are ahead in the race as dentists, should have been glad of the opportunity of lending a helping hand by our presence and action. If dentistry needs the cultivation of art, with the science which has been achieved, it would be more apt to receive it from France; for surely she stands at the head of the world in all that is grand and beautiful in art and sculpture, and to aid such a people in our peculiar practical way would have not been lost to us. Besides, we need not feel that we are the only ones who have done anything for dentistry. When the French once get a start under their new Republican principles, we shall see an advance in every line that will startle us. Hence, I say, "Vive le First International Dental Congress," for the good work it did in arousing the dentists of Europe.

As to whether there should be a second congress is a matter to be discussed. Now that the physicians have given us a section, it is well we should hold to this, at least until we become so unwieldy a body that we shall have to stand alone. The advantages of our present association with medicine is surely something. To have been recognized as worthy of the attachment to the main body, establishes the fact that we have done it for ourselves, and not that we are so much the gainers as they. The French will not have their pride touched should there not be another similar meeting anywhere else. They did a good thing, and they will feel and see its influence in the future, and there was not a man at their meetings who regrets he participated.

As to the social side of the congress, it was equal to anything to which I was ever invited. Their banquet as well as their private entertainments had an enthusiasm and spirit shown that would be well for us to imitate. They surely believe in enjoying life to the full.

The cases at the Exposition of cleft palate and various restorations of the face were the largest collection and finest I ever saw. In this work they excel. For some reason they have more of such operations than we have, and they lead us here. I saw one case in the private practice of Dr. Michaels where all of the superior maxilla to near the orbit of the eye, and as far up and back as possible to reach without involving the main arteries and the whole of the inferior jaw, had been completely removed by one of the celebrated French surgeons. It was a frightful case. I have here photographs of the woman before and after the restoration by Dr. Michaels, of Paris, who was successful in placing in not only a nose and upper lip, but a set of teeth for both jaws, so far as appearances go, but not for actual use, as it was impossible for him to take an impression. He had to fit the whole thing in by individual pieces. The appearance was all that could be asked, but it necessitated a great deal of trouble to remove the apparatus and cleanse it.[1]

---

[1] In further justice to Dr. Michaels, I must mention his new style of gold or silver plates for artificial teeth, which was rather unique.

Clasped plates, as small as possible, are the rule on the continent; and, to enable the operator hastily to form a plate without getting up dies, he conceived the plan of having twenty-two-carat gold rolled into sections or blocks of one-sixteenth of an inch square, so that the blocks were held together by not being cut entirely through, leaving the plate very thin between each section. With a cloth, or the finger alone, this could be pressed on to the surface of the plaster cast and take its form quite perfectly. The clasps and teeth are now adjusted on to the cast, and the plate held down on the plaster cast with wire. Twenty-carat solder is run on the surface of the plate, which fills up between the blocks, and makes a solid plain surface and quite stiff. The plaster cast has as much sand mixed with it as is used in making the usual investment for soldering. For cheap and hasty work it is good. The great objection with me would be that, as I never solder the clasps to the plate from the plaster model

One very novel instrument that I saw was a pair of forceps, at one of the schools, invented by one of the professors. It was on the same principle as a monkey-wrench, with the regular jaws of forceps, which enabled the operator to hold the jaws or beaks in close contact with the tooth to be extracted, and there was no possibility of crushing the tooth, nor slipping off, from grasping too tightly.

Compressed air and electricity are playing quite a part in many of the offices in running all their machinery, and aiding in many ways to secure better results with less expenditure of force. My own clinics were in gold and amalgam filling, and the use of the articulator in making artificial teeth. It was a surprise to all to see perfectly smooth oval pluggers wiping in gold at the rate of one book and a half in forty-three minutes; also how it was possible to make Abby's old-fashioned gold foil adhere as perfectly as any of the most cohesive foil. Compared with the slow packing of gold this was to them a revolution, and they could well see and express the belief that it was now possible for them to begin to use gold as much as we do, and get results never before attained.

When I spoke before the congress of my articulator and its uses and the importance of the geometrical laws concerned in studying cases of irregularity, Dr. Davenport read the following, which I feel in justice to myself should be repeated:

"The long observation and study I have given the articulation of the teeth, with the hope of determining the laws governing their relations to each other and to the surrounding parts, anatomically, physiologically, and mechanically, enable me to appreciate with the keenest delight what Dr. Bonwill has so beautifully explained to us. He has expressed a most intense practicality in the very highest scientific terms.

"Those observers who object to ideal standards of comparison, and insist upon average conditions as the only safe basis, ought

---

on account of making an imperfect fit, I fit the plate to mouth first, then the clasps to teeth in the mouth, and while the plate is held in position I place plaster over the clasps and plate, and allow it to harden, and when removed from the mouth run into this plaster and sand and solder. This will always make perfect adjustment, and there will be no wear on the teeth and the clasp can be very loose. To do this with Dr. Michaels's plate, it would have to be soldered over its face first, and then the teeth and clasps afterwards. I saw this same thing at Louisville, Ky., in 1888, but made from fine twenty-two-carat wire gauze or netting, and the meshes afterwards filled in with solder. I think this would make a better adjustment. This is only intended for partial sets; it will not do for suction.

to close their mouths until the pure science first taught by Dr. Bonwill penetrates their ears, and their eyes have seen its practical results.

"As a profession we ought to confess to Dr. Bonwill our regret that the meaning of his words uttered thirty years ago are only now being comprehended by us. To-day, the profession, in reopening an old mine, has uncovered a priceless gem which once was thrown aside as worthless."

I had previously explained, on several occasions, to him and several scientific gentlemen whom he had invited to meet me, until he had become very thoroughly imbued with its importance, as the lines just read plainly indicate.

I do not know whether you will consider it any honor to you for your delegate to have had any special favors shown him at the congress; but, as it was done, and as no one else received a like ovation, and as it comes to you in the report of their own journals, you cannot doubt it. They could not have shown more consideration for any hero, and it was done unstintedly and on all occasions.

I trust there will be no misunderstanding of what I have said by any of my good friends on the "other side," for I have sedulously endeavored to impress my countrymen with the facts as I have received them. Something should be done to harmonize the status of dentistry in all parts of the world, so that each man can feel that there are no lines of nationality; that the sole aim is the greatest good to the greatest number. We must always expect more or less of contention, for the medical profession, after three thousand years, has the same to face everywhere. The native practitioner is always jealous and envious of the foreigner wherever he may be. We have it here, rampant, in every hamlet. The best way to eradicate it is to make ourselves competent, and so far above the competition as not to fear his inroads. This must lead the weaker one a step higher also.

As we appear to have so much in our hands that is calculated to advance the interests of dentistry, and make us all more akin throughout the world, I would advise that we do all we can to make of us a profession that knows no nationality, that every man, in whatever nation he may be born, educated, or may practise, should feel the assurance of being well received here, and that, when any of us go abroad to practise, we shall do nothing under the title of "American dentist."

DISCUSSION OF DR. E. P. WRIGHT'S PAPER ON "BLEACHING."

*Dr. E. C. Kirk.*—I feel that the thanks of the society are due to Dr. Wright for the trouble he has taken in coming to our meeting and giving us the valuable and practical points which he has presented in his paper. I am especially interested in one point he has made,—viz., the necessity for the complete removal of all fatty matter from the tooth-structure previous to the application of the bleaching agent. While I have not experienced great difficulty in restoring teeth of a bluish tint to a normal color, I have found—and I have no doubt that it is the common experience of all who have made the attempt—that it is well-nigh impossible to get satisfactory results in bleaching teeth which are of a dark yellow or brown tint; and this result obtains regardless of the agent or method employed, for, I think, I have tried them all. It may be—and indeed it seems likely from the experiment reported by Dr. Wright—that a complete removal of all fatty matter by the method he suggests, or one of a similar nature, may prove to be the solution of the difficulty in these obstinate cases.

A special point made by the speaker, and one which cannot be too strongly emphasized, is the necessity for continuing the treatment until the bleaching agent has penetrated to the ultimate ends of the dental tubuli, otherwise a recurrence of discoloration will result. With regard to the choice of use between dry chlorine and its aqueous solution, it is well to bear in mind that, in general, chlorine bleaches best in the presence of moisture. It does not act directly on the coloring matter, but indirectly, by virtue of its affinity for hydrogen. In the presence of moisture it seizes upon the hydrogen of the water molecule, liberating its oxygen, which in its nascent state is the active bleaching agent.

*Dr. Head.*—When the fatty matter is to be removed, is your cavity moist, or dry?

*Dr. Wright.*—I do not think it of much importance whether it is wet or dry. The obstruction to the passage of the chlorine water, from the centre to the periphery, must be removed. This I believe to be the fatty matter. The other day I took some hygrometric material, placed it around a tooth, exhausted the native air by the piston, and put in four or five drops of ether, which, with the loss, amounted to perhaps two drops, and by means of the chlorine the tooth was made beautiful and white. The apparatus which has been shown is simply a means of putting the tooth in a vacuum.

The most important part is the removal of the fatty matter; after this is done, pack your cavity with cotton saturated with distilled water, and with your syringe inject the chlorine gas, and you have chlorine water at work.

*Dr. Head.*—I know if you drop a dry tooth in ink it will be colored only on the outside; but if the tooth be previously wet with water, it will be colored through and through. I thought this might make some difference in the action of the chlorine.

*Dr. Woodward.*—Is the apical foramen open, or how do you fill it?

*Dr. Wright.*—At the apex I put gold to one-third the distance; then gutta-percha; scrape out as much dentine as the tooth will allow, and then proceed. Always scrape out a little more than you think is enough.

*Dr. Sudduth.*—Dr. Wright has given us clinically what I have demonstrated microscopically in the laboratory, and my observations coincide perfectly with his theory. The histology of the part, as I have demonstrated for fifteen years, shows that coloring-matter can be extended along the living fibres. On the other hand, I have taken a tooth and made a section of it, immediately following an inflammation of the pulp, and found a condition of fatty degeneration in the dentinal fibrillæ. When this is present the finest tincture will not succeed in coloring the periphery of the fibres; but when the fat is removed by ether, the staining shows to the very outside.

*Dr. Truman.*—I confess to being much interested in the paper and the remarks of Dr. Wright. I think his process marks an advance, and should be permanently brought forward, so that all may become familiar with it. I imagine very few here have a practical idea of its form or application. I have had so much to do with bringing this subject before the profession, and have had to combat so frequently unthinking prejudice, that I feel disinclined to say more upon it.

Like all other processes, it has had its period of origin and subsequent development, its time of crude ideas and more perfected modes; but the education of dental thought in this direction has not kept pace with improvements.

The professional mind is no more prepared to-day to believe that the bleaching of teeth is a part of operative dentistry than it was twenty-five years ago, when I first attempted to prove its importance. This is due, doubtless, to many reasons; but prominently to the one fact that but few will observe the rules laid down for their government; and, consequently, the result does more harm than good, the process shouldering the responsibility of the failure. Hence I welcome an apparatus that eliminates, to a large degree, the personal equation.

The use of chlorine for bleaching is not new; indeed it is the oldest process; but its irritating effects made it impossible to use. Dr. Wright's apparatus overcomes this entirely, and hence my reason for some degree of faith in it. I have not seen it in operation but in one case, and that was one of the worst possible. This came into my hands for treatment after it had been tried by some inexperienced operator. I found it entirely black. Suspecting the cause of this, I proceeded to bleach in the usual way, and soon returned the tooth to a deep yellow tinge. This the patient reported as the original shade. Satisfied that this color could not be changed, I handed the case to a professional friend to try his plan. This resulted the same way. Dr. Wright, happening in the city at the time, made a fourth attempt, and the temporary result was remarkable; but, unfortunately, it failed as the others had done. This case is cited in detail not to antagonize the process, but to emphasize the difficulties that must surround all attempts at bleaching, and the importance of giving more attention to it. We have yet to learn whether speedy bleaching by Dr. Wright's process will be as lasting as by slower modes. If this proves to be the case, then there is no question but that we have in this plan a more effective mode than has heretofore been brought to the notice of the profession, and one that is, at the same time, so easily applied and is so positive in its action, that it would seem to have a future of usefulness before it. I, however, do not believe it or any other process will change the yellow color of teeth. These constitute but a small proportion, the blue predominating.

*Dr. Jameson.*—Can one-half or one-third be bleached?

*Dr. Wright.*—More.

*Dr. Tees.*—How often is the apparatus used before the bleaching is done?

*Dr. Wright.*—Only once.

*Dr. Tees.*—How about the tooth of which Dr. Truman spoke?

*Dr. Wright.*—As I said before, there are some teeth that cannot be bleached; and I think the doctor admitted that this particular tooth had resisted all other efforts to bleach it. But I find that ninety-five per cent. of all teeth may be bleached.

*Dr. Tees.*—Will these ninety-five per cent. remain white long?

*Dr. Wright.*—I can show you cases that have been bleached more than eighteen months, and they are just as white now as when first done.

Subject passed.

INCIDENTS OF PRACTICE.

*Dr. Place.*—A young lady came to me suffering periodically with severe pain through the nerve which supplies the first and second lower bicuspids on the right side. She had been suffering for over a week. It comes every half-hour or fifteen minutes, and lasts five or ten minutes. While it lasts it is excruciating. A careful examination of the mouth showed the wisdom-tooth and second molar in place, the sixth-year molar having been extracted years ago. In the wisdom-tooth as well as the twelfth-year molar there were large amalgam fillings. The bicuspid teeth are entirely sound, with no sign of decay between them. I took an instrument and tapped it lightly on the wisdom-tooth, also on the twelfth-year molar, and in neither could I get a response. As soon as I tapped the second bicuspid she felt a slight pain. I applied the rubber dam on the wisdom-tooth, to see if the nerve was alive, and found it was; likewise that in the twelfth-year molar and that in the bicuspid,—all alive. I hardly knew what course of treatment was indicated. I applied to the gums arsenic and aconite, and she is now under the care of a physician, who, I suppose, is treating her with quinine. If there is better treatment I would like to use it. By pressing on the nerve at the mental foramen, there is a tendency to relieve the pain. It was used several times when the pain was very severe, which gave temporary relief.

*Dr. James Truman.*—Are the teeth very dense? If so, there might be granular deposits in the pulp. I think I should drill into one of these bicuspids.

*Dr. Bassett.*—Is it not possible that this trouble comes from the wisdom-tooth,—from the amalgam filling? The trouble might be reflected forward into the bicuspid. I have seen one or two cases where this has been the result, and it may be so with this case.

*Dr. Kirk*—I think the fact of cold relieving the bicuspid precludes the idea of the trouble arising from pulp granules.

*Dr. Guilford.*—As there are several points in Dr. Bonwill's paper about which I should like to speak, and which I think others here would like to discuss, I move that the discussion be postponed until our next meeting, owing to the lateness of the hour. Carried.

The meeting then adjourned.

